# Expansion of Vaccination Services and Strengthening Vaccine-Preventable Diseases Surveillance in Haiti, 2010–2016

**DOI:** 10.4269/ajtmh.16-0802

**Published:** 2017-10-18

**Authors:** Rania A. Tohme, Jeannot Francois, Kathleen F. Cavallaro, Gilson Paluku, Idrissa Yalcouye, Ernsley Jackson, Tracie Wright, Paul Adrien, Mark A. Katz, Terri B. Hyde, Pape Faye, Francine Kimanuka, Vance Dietz, John Vertefeuille, David Lowrance, Benjamin Dahl, Roopal Patel

**Affiliations:** 1 Global Immunization Division, Centers for Disease Control and Prevention, Atlanta, Georgia;; 2 Direction du Program Elargi de Vaccination (DPEV), Ministry of Public Health and Population, Port-au-Prince, Haiti;; 3 Pan American Health Organization, Port-au-Prince, Haiti;; 4 United Nations Children’s Fund, Port-au-Prince, Haiti;; 5 Direction d’Épidémiologie, de Laboratoire et de Recherche (DELR), Ministry of Public Health and Population, Port-au-Prince, Haiti;; 6 Centers for Disease Control and Prevention, Port-au-Prince, Haiti

## Abstract

Following the 2010 earthquake, Haiti was at heightened risk for vaccine-preventable diseases (VPDs) outbreaks due to the exacerbation of long-standing gaps in the vaccination program and subsequent risk of VPD importation from other countries. Therefore, partners supported the Haitian Ministry of Health and Population to improve vaccination services and VPD surveillance. During 2010–2016, three polio, measles, and rubella vaccination campaigns were implemented, achieving a coverage > 90% among children and maintaining Haiti free of those VPDs. Furthermore, Haiti is on course to eliminate maternal and neonatal tetanus, with 70% of communes achieving tetanus vaccine two-dose coverage > 80% among women of childbearing age. In addition, the vaccine cold chain storage capacity increased by 91% at the central level and 285% at the department level, enabling the introduction of three new vaccines (pentavalent, rotavirus, and pneumococcal conjugate vaccines) that could prevent an estimated 5,227 deaths annually. Haiti moved from the fourth worst performing country in the Americas in 2012 to the sixth best performing country in 2015 for adequate investigation of suspected measles/rubella cases. Sentinel surveillance sites for rotavirus diarrhea and meningococcal meningitis were established to estimate baseline rates of those diseases prior to vaccine introduction and to evaluate the impact of vaccination in the future. In conclusion, Haiti significantly improved vaccination services and VPD surveillance. However, high dependence on external funding and competing vaccination program priorities are potential threats to sustaining the improvements achieved thus far. Political commitment and favorable economic and legal environments are needed to maintain these gains.

## INTRODUCTION

The World Health Organization (WHO) has estimated that about 1.5 million children less than 5 years of age continue to die annually from vaccine preventable diseases (VPDs) globally; these deaths constitute approximately 20% of overall childhood mortality.^1^ Vaccination is considered the first line of public health defense and one of the most efficient and cost-effective public health interventions for reducing childhood illness and mortality. High vaccination coverage can reduce the proportion of people who are susceptible to infection with VPDs and can interrupt their transmission and spread. Furthermore, an effective national vaccination program decreases the potential for spread of VPDs to other countries.

The region of the Americas has made great progress in preventing VPD morbidity and mortality, by eradicating polio in 1994,^2^ interrupting endemic measles and rubella transmission since 2002 and 2009,^3^ respectively, and eliminating maternal and neonatal tetanus from all countries except Haiti.^4^ Following the 2010 earthquake, however, long-standing problems in the vaccination program in Haiti were exacerbated, and routine immunization coverage has remained particularly low.^5^ At the same time, large numbers of people came to Haiti to provide humanitarian aid and other support activities from multiple countries, including countries where polio, measles, and rubella were endemic. Those events put Haiti at high risk of VPD importation and outbreaks. Therefore, a key component of postearthquake public health reconstruction efforts in Haiti was building a more robust national vaccination program, involving public, private, and nongovernmental institutions. Subsequently, many international organizations partnered with the Haiti Ministry of Public Health and Population (French acronym: MSPP) and the Directorate of the Expanded Program on Immunization (French acronym: DPEV) to strengthen immunization services and improve detection and response to VPDs.

This article highlights key activities that were implemented in Haiti during 2010–2016 to maintain the country free of polio, measles, and rubella, and achieve maternal and neonatal tetanus elimination (MNTE). In addition, we report on the expansion and improvement in cold chain capacity and vaccine management and on the introduction of new and underutilized vaccines to reduce under five and overall mortality in Haiti. Furthermore, we summarize the main improvements in VPD surveillance, which were implemented to ensure rapid detection and response to outbreaks, and we evaluate the impact of the newly introduced vaccines. Finally, we discuss priorities needed to sustain those achievements.

## ELIMINATING MEASLES, RUBELLA, AND TETANUS, AND MAINTAINING POLIO ERADICATION

### Measles, rubella, and congenital rubella syndrome elimination.

Measles vaccine was introduced in Haiti in 1982. In November 2007, Haiti was the last country in the Americas to introduce the rubella-containing vaccine through a nationwide vaccination campaign that targeted persons aged 1–19 years, and in 2008, the vaccine was introduced in the national vaccination schedule.^6^ The current routine vaccination schedule includes one dose of measles–rubella (MR) vaccine at the age of 9 months. During January 2000 to September 2001, over 1,000 confirmed measles cases were reported in Haiti.^7,8^ The outbreak was controlled by supplementary immunization activities (SIAs) implemented throughout the country using fixed posts and door-to-door vaccination.^7^ The last cases of measles and rubella in Haiti were reported in 2001 and 2006, respectively. In 2007, member states of the Pan American Health Organization (PAHO) passed a resolution to document and verify the interruption of endemic measles, rubella, and congenital rubella syndrome (CRS) in the Americas.^9^ Strategies to achieve and maintain elimination of endemic measles and rubella included achieving high (≥ 95%) two-dose vaccination coverage with MR vaccine, and having a surveillance system capable to detect, investigate, and respond to all suspected cases.^3^

**Figure 1.**
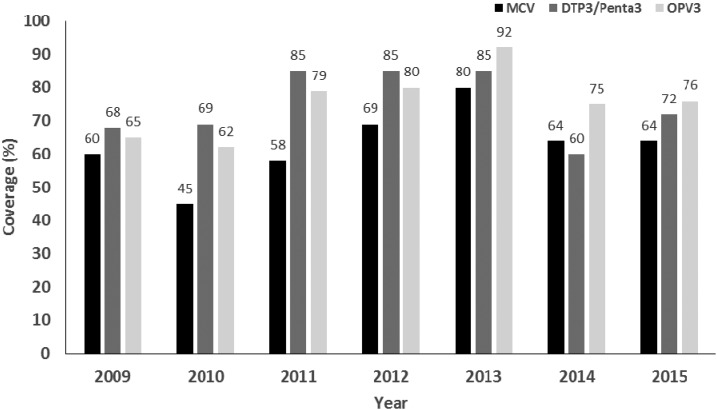
Coverage with three doses of diphtheria, tetanus, pertussis vaccine (DTP3) and oral polio vaccine (OPV3), and one dose of measles-containing vaccine (MCV) among children 12 months of age—Haiti, 2009–2015. Source: WHO.^5^

Before 2012, reported administrative coverage in Haiti with one dose of measles-containing vaccine (MCV) among infants aged 12 months had been below 60% (Figure 1),^5^ and surveillance for measles and rubella relied mainly on passive reporting. To prevent measles outbreaks after the 2010 earthquake, a large vaccination campaign was implemented during February–June 2010 in internally displaced persons camps, and additional vaccination activities were conducted during “child health days” in November 2010, achieving a coverage of over 80% among children aged < 5 years.^10,11^

In 2012, to maintain and verify measles, rubella, and CRS elimination, DPEV implemented a combined MR and oral polio vaccine (OPV) vaccination campaign targeting children aged 9 months to 9 years. During the campaign, active community-based and health facility-based surveillance for suspected measles, rubella, and CRS cases was conducted, and a retrospective review of hospital records for CRS cases was undertaken.^12^ A vaccination coverage survey conducted shortly after the campaign found that 91% of children aged 1–9 years had received at least one dose of MCV in their lifetime, and 82.2% (95% confidence interval [CI]: 80.2–84.1%) of children received MR during the 2012 campaign. Furthermore, during the 2012 campaign, 51% of children aged 1–9 years received their second MR dose, whereas 32% received MR for the first time.^13^ Active surveillance and retrospective review of hospital records did not identify suspected cases of measles, rubella, and CRS, indicating that Haiti had potentially eliminated those diseases. A serosurvey conducted in 2012 among pregnant women attending antenatal care clinics showed that 94% and 96% of women aged 15–39 years were immune to measles and rubella, respectively.^14^ As a result of the MR campaign and enhanced surveillance activities implemented in 2012, the International Expert Committee for the verification of measles, rubella, and CRS elimination in the Americas certified in 2014 that Haiti had eliminated those diseases. In 2015 and 2016, PAHO declared that the Americas had interrupted endemic transmission of rubella and measles, respectively, becoming the first region globally to eliminate measles and rubella.^15,16^

Despite the success in eliminating measles, rubella, and CRS, Haiti needs to maintain elimination by ensuring a high (> 95%) routine vaccination coverage with MR vaccine. During 2014–2015, coverage with MR decreased to less than 70% (Figure 1) due to vaccine stock-outs and competing priorities for staff in health facilities and departments, who were responsible not only for the provision of routine vaccination services but also the implementation of cholera and tetanus vaccination campaigns. To address the low routine MR vaccination coverage, DPEV implemented an integrated MR and OPV vaccination campaign in March–April 2016, targeting almost 1.5 million children aged 9–59 months. The administrative MR coverage achieved during the campaign was estimated at 99% (DPEV, personal communication).

Despite the successes of these vaccination campaigns in increasing population immunity, campaigns should not replace routine services, which remain the main building blocks of a strong and sustainable vaccination program. To address immunity gaps for measles, Haiti should consider adding a second dose of measles vaccine after the age of 12 months.

### Maternal and neonatal tetanus elimination.

In 2012, Haiti was the only country in the Americas that has not yet achieved MNTE. MNTE is defined as less than one case of neonatal tetanus per 1,000 live births per district per year.^17^ It is estimated that about 50% of all neonatal tetanus cases reported annually in Latin America and the Caribbean are from Haiti. Before 2012, Haiti had low coverage with two or more doses of tetanus–diphtheria vaccine (Td2+) among pregnant women (< 65%)^18^ and with three doses of diphtheria, tetanus, and pertussis vaccine (DTP3) among children (range: 60–85%).^5^ In addition, according to the 2012 Demographic and Health Survey (DHS), only 38% of births occurred in health facilities; thus, nearly two-thirds of Haitian children were born at home with varying and unregulated levels of medical assistance or clean delivery.^18^

To move forward with MNTE, in 2013, Haiti developed and implemented a plan to eliminate MNT by 2015, which included the following objectives: 1) vaccinate at least 80% of women of childbearing age through three rounds of SIAs; 2) sustain routine immunization coverage with DTP3 at ≥ 80% among children under 12 months of age, and reduce the DTP1 to DTP3 dropout rate to < 10% in all communes; 3) improve the quality of deliveries by training midwives and traditional birth attendants, expanding available equipment for deliveries, and strengthening social mobilization; and 4) strengthen epidemiological disease surveillance to detect neonatal tetanus cases.

The MNTE 2013–2015 plan aimed to vaccinate a total of 2,212,055 women of childbearing age (15–49 years) living in all 140 communes of Haiti with three doses of Td vaccine. Three rounds of Td vaccination campaigns were implemented in April, May, and November 2013 in 65 high-risk communes, and the remaining 75 communes were targeted with three rounds of Td vaccination in 2014. In 2015, mop-up vaccination campaigns were conducted in communes with low coverage (Td2+ < 80%). The overall Td2+ coverage achieved during the 2013–2015 SIAs was 87%. In total, after those campaigns, 94 (67%) of 140 communes in the country achieved a Td2+ coverage above 80%, 31 (22%) communes had a Td2+ coverage of 50–80%, and 15 (11%) had a Td2+ coverage < 50%. Furthermore, in 2015, a pilot community-based surveillance project was implemented to strengthen surveillance and detection of neonatal tetanus.

In July 2016, a pre-validation assessment was completed and concluded that the country is eligible for the final validation assessment. In August 2017, a validation survey verified that Haiti has eliminated maternal and neonatal tetanus.

### Maintaining polio eradication.

In 1994, the Americas was the first region to be declared free of wild poliovirus (WPV). However, until global eradication is achieved, children living in the region could still be infected with polio either through importation or through circulating vaccine-derived polio virus (cVDPV) infection. Countries where there is low polio vaccine coverage are at increased risk for polio cases or outbreaks because higher proportions of their populations are susceptible to infection. To maintain polio-free status and detect and respond to potential outbreaks in a timely manner, Haiti should maintain an OPV3 vaccination coverage ≥ 95% in all districts and meet required surveillance indicators for acute flaccid paralysis (AFP).^2^

Although OPV3 coverage in Haiti has been improving, it is still below the recommended coverage needed to maintain immunity in the population (Figure 1). During 2000–2001, a total of eight confirmed vaccine-derived polio cases (type 1), including two fatal cases, were reported in Haiti.^19^ Of these, only one child had documentation of three doses of OPV; the other children were either unvaccinated or incompletely vaccinated. To raise population immunity to polio in Haiti, combined MR and OPV campaigns were conducted in 2012 and 2016, as described in the Measles, Rubella, and CRS Elimination section.

In January 2016, following the Global Polio Eradication Initiative recommendations,^20^ Haiti introduced one dose of inactivated polio vaccine (IPV) at the age of 6 weeks in its routine vaccination schedule, and in May, the country switched from using trivalent to bivalent OPV. Although no wild or vaccine-derived poliovirus cases have been reported in Haiti during the past decade, the country remains at increased risk for polio outbreaks if routine coverage is not improved.^21^

## Cold chain expansion and strengthening vaccine management

### Expansion of the vaccine cold chain.

A national vaccination program’s cold chain system includes temperature-controlled storage units and transport equipment, including cold rooms, freezers, refrigerators, cold boxes, and vaccine carriers.^22^ Cold chain systems ensure that vaccines and diluents are warehoused, disseminated, and delivered within the recommended temperature ranges for all categories of vaccines.^22^ The vaccine cold chain in Haiti consists of three levels. The national level consists of a storage warehouse in Port-au-Prince, which houses all the vaccines intended for the country. Vaccine storage at the national level is accommodated by walk-in electric-powered negative (< 0°C) and positive temperature (+2°C–8°C) cold rooms. The intermediate level consists of vaccine depots in each of the 10 departments in the country and in some of the large communal sections in departments with difficult geographic access (Center, Nord-Ouest) or a large population (Artibonite, Ouest). The intermediate level includes electric- and solar-powered refrigerators. The peripheral level cold chain consists of a mix of propane, solar, and electric refrigerators located in health facilities that provide vaccination.

Shortly after the earthquake, international immunization partners including Centers for Disease Control and Prevention (CDC), PAHO, and United Nations Children’s Fund (UNICEF) assisted DPEV in assessing and strengthening the cold chain. Assessments of the cold chain capacity at various levels were conducted in 2011, 2013, and 2015 to determine the country’s capacity for positive temperature cold storage, and to estimate the additional capacity required to accommodate the introduction of three new vaccines (pentavalent, rotavirus, and pneumococcal conjugate vaccine [PCV]). Based on those assessments, cold chain capacity was expanded.

Three positive-temperature cold rooms were added at the national level during 2012–2014, which increased the capacity of storage by 91% compared with 2011 and provided 103% of the required capacity to store traditional vaccines (Bacillus Calmette–Guérin [BCG], OPV, MR, Td), and pentavalent, pneumococcal, and rotavirus vaccines as determined by the 2015 needs assessment (Table 1). At the intermediate level, 70 high-capacity solar refrigerators (each having a vaccine storage capacity of 156 L) were added during 2013–2014. These additions increased the overall vaccine storage capacity at the intermediate level by 285% compared with 2011 and provided 100% of the required capacity in 2017 as determined by the 2015 assessment (Table 1).

**Table 1:** Required and available positive temperature cold chain storage capacity based on cold chain assessments—Haiti, 2011–2015

	Capacity required (L)			Capacity available (L)			Percent of required capacity		
Year of cold chain Assessment	2011*	2013†	2015‡	2011	2013	2015	2011	2013	2015
National	50,000	35,445	31,499	17,000	26,923	32,500	34	76	103
Intermediate	8,800	14,129	14,539	3,814	3,702	14,704	43	26	101
Peripheral	ND	9,099†	ND	ND	32,056	ND	ND	352	ND

BCG = Bacillus Calmette–Guérin; MR = measles–rubella; OPV = oral polio vaccine; ND = not determined; Td = tetanus–diphtheria.

* Cold chain capacity assessment 2011 estimated capacity required in 2017 for traditional vaccines (BCG, OPV, MR, Td) and pentavalent, pneumococcal, and rotavirus vaccines, based on projected national population through 2017 and four vaccine shipments per year.

† Cold chain capacity assessment 2013 estimated capacity required in 2014 for traditional vaccines and pentavalent, pneumococcal, and rotavirus vaccines, based on available population data for municipalities, or if not available, on average municipality population.

‡ Cold chain capacity assessment 2015 estimated capacity required in 2017 for traditional vaccines and pentavalent, pneumococcal, and rotavirus vaccines (after replacing the rotavirus vaccine having the syringe applicator [85.3 cm^3^/dose] with the vaccine having a tube applicator [17.1 cm^3^/dose]), based on estimated population data.

Although the capacity of the peripheral level was more than adequate in 2013, the distribution of refrigerators across heath facilities was suboptimal and some health facilities could not provide vaccination services because of inadequate cold chain capacity or shortages in propane gas. Since 2015, a plan has been implemented to ensure adequate distribution of refrigerators, gradually replace propane-powered with solar-powered refrigerators, and repair non-functional solar refrigerators at the peripheral level. In 2014–2016, a total of 111 solar refrigerators were installed at the peripheral level and have been documented to work properly for vaccine storage. During the next 3 years, with funding support from Gavi, the Vaccine Alliance, Haiti, is expected to install 721 solar direct drive (SDD) refrigerators for vaccine storage, mostly at the peripheral level. These SDD refrigerators will gradually replace the propane-powered refrigerators, which no longer meet the standards for vaccine refrigerators established by WHO.

### Improving vaccination supply chain and effective vaccine management.

A good vaccination supply chain and effective vaccine management (EVM) are essential for immunization programs.^23^ Haiti has regularly experienced frequent vaccine stock-outs at various levels with shortages sometimes lasting several months. To identify obstacles and propose solutions to improve the vaccine supply chain and management in Haiti, DPEV with support from PAHO and UNICEF, conducted an EVM evaluation in 2013. The evaluation found that standard operating procedures (SOPs) for cold chain maintenance, vaccine transport and distribution, and emergency plans for vaccine storage had not been established; the electronic stock management tool was not being used; and there was a mismatch between inventory records and actual vaccine stock availability. In addition, the immunization program lacked written instructions on requisitioning vaccines and supplies, up-to-date estimates and justification of vaccine quotas for the department and health facility levels, and real-time monitoring of the stock of vaccines and immunization supplies.

To improve vaccine management, during 2013–2015, cold chain staff from the central and department levels were trained on the Vaccination Supply Stock Management (VSSM) system. VSSM is a vaccine management system developed by WHO to help users manage, document, and track the receipt, storage, and distribution of vaccines, syringes, and other immunization supplies.^24^ VSSM software was installed on computers in the central vaccine warehouse and in all departmental depots in Haiti. As a result of implementing VSSM, DPEV was able to 1) electronically monitor receipt, storage, and distribution of vaccines and supplies from the central level to each department intermediate depots; 2) adjust quarterly provisions to departments’ intermediate depots and institutions based on updated quotas and real-time stock levels; and 3) execute, distribute, and respond to monthly vaccine and supply reports. DPEV staff subsequently worked with partners to develop SOPs for the requisition of vaccines and supplies and for maintenance of cold chain equipment.

Although availability of vaccines and sufficient cold chain capacity to store vaccines are essential to improve vaccination services, it is also important to store vaccines at the appropriate temperature to ensure adequate potency. To monitor the temperature of newly installed solar refrigerators and ensure adequate function, in 2014, remote data loggers (RDLs) were installed in 15 sites (11 sites in Ouest and four sites in Artibonite Departments). RDLs record the interior and exterior temperatures of refrigerators and the refrigerator door status (open/close). This project also explored the feasibility of obtaining useful data from RDLs for vaccine cold chain monitoring and documented RDL installation and operations costs. The temperature monitoring devices sent immediate notification via text messages to project personnel whenever the temperatures went outside the allowed range for an extended time period (Kathy Cavallaro, personal communication). As a result, immunization staff were able to immediately respond to temperature aberrations to keep vaccines at recommended temperatures.

## INTRODUCTION OF NEW AND UNDERUTILIZED VACCINES

Respiratory infections and diarrheal diseases are among the leading causes of infant mortality in Haiti. According to the 2012 DHS, 14% and 21% of children under 5 years of age had an acute respiratory infection or a diarrheal episode during the 2 weeks prior to the interview, respectively.^18^ A retrospective review of hospital admission registries during January 1, 2011 to December 31, 2013, revealed that respiratory diseases accounted for 29% of hospitalizations and 17% of deaths, and diarrheal diseases accounted for 26% of hospitalizations and 13% of deaths among children less than 5 years of age in Haiti.^25^ Children aged 6–23 months had the highest percentage of hospitalizations attributable to respiratory diseases (38%). Children aged 6–11 months had the highest percentage of diarrhea-associated hospitalizations (39%). Diarrheal disease admissions peaked during the dry season in January–April, which is similar to trends in rotavirus disease seen in other tropical countries.^25^

Given the high burden of respiratory and diarrheal diseases among infants in Haiti, DPEV, with financial support from Gavi and CDC, introduced pentavalent vaccine that protects against five diseases (diphtheria, tetanus, pertussis, hepatitis B, and *Haemophilus influenzae* type b [Hib]) in 2012 and rotavirus vaccine in 2014. DPEV is also planning to introduce PCV-13 in the near future. These three vaccines have the potential to reduce mortality considerably in Haiti. Using estimates of annual deaths in Haiti from *H. influenzae* type b,^26^ hepatitis B virus (HBV)-related liver cancer and chronic liver disease,^27^ rotavirus diarrhea,^28,29^ and pneumococcal pneumonia,^26^ estimates of vaccine effectiveness,^27,30–32^ and reports of current pentavalent and rotavirus vaccine coverage in Haiti,^5^ we assessed the number of deaths in Haiti that could be prevented by pentavalent, rotavirus, and PCV-13 introduction (Table 2). Overall, the three vaccines could prevent 5,227 deaths per year in Haiti. Hepatitis B vaccine is estimated to have the biggest impact by potentially preventing 2,480 deaths in the overall population. Hib vaccine, rotavirus vaccine, and PCV-13 could together prevent an estimated 2,747 deaths among children under 5 years of age (Table 2).

**Table 2:** Estimated number of annual deaths that could be prevented by pentavalent, rotavirus, and pneumococcal conjugate vaccines in Haiti

Vaccine	Estimated annual deaths (95% CI) [A]	Estimated vaccine effectiveness (range) [B]	Estimated annual deaths preventable by vaccination (95% CI) [C]*	Estimated vaccine coverage in Haiti (%) [D]^5^	Estimated vaccine preventable deaths in Haiti (range) [E]†
Hib	1,565^26^ (906–2,203)	95%^30^	1,487 (861–2,093)	75	1,115 (646–1,570)
Hepatitis B	3,480^27^	95%^27^	3,306	75	2,480
Rotavirus	800 (710–889)^28^	60% (50–70%)^31^	480 (426–533)	60	288 (256–320)
PCV	2,987^26^ (2,061–3,287)	60% (50–70%)^32^	1,792 (1,237–1,972)	75	1,344 (928–1,479)
Total	**8,832**	**–**	**7,065**	**–**	**5,227**

CI = confidence interval.

* [C] = [A] × [B].

† [E] = [C] × [D].

Despite recommendations by the Strategic Advisory Group of Experts on immunization to provide a dose of hepatitis B vaccine within 24 hours of birth,^33^ Haiti has not yet included a birth dose in the immunization schedule. In 2012, a hepatitis B serosurvey was conducted among a representative sample of pregnant women attending antenatal care clinics to estimate the burden of chronic HBV infection and the risk of mother-to-child transmission of HBV.^34^ Results showed that 2.5% of pregnant women had chronic HBV infection; almost half (46%) of pregnant women with chronic HBV infection had high viral loads (HBV DNA > 5,000 IU/mL), and 27% had very high viral loads (HBV DNA > 200,000 IU/mL), which greatly increase the risk of transmission of HBV infection from the mother to the child during delivery.^34^ These data underscore the need to introduce a birth dose of hepatitis B vaccine in Haiti.

## STRENGTHENING VACCINE PREVENTABLE DISEASES SURVEILLANCE AND OUTBREAK RESPONSE

Strong surveillance systems able to detect, investigate, and respond to VPDs are essential to ensure that polio eradication and measles, rubella, and CRS elimination are maintained, and to verify MNT elimination. A sensitive surveillance system is needed for a timely response to any VPD outbreak and to prevent spread of infectious diseases in the country. In addition, surveillance is essential to understand the epidemiology and burden of diseases and to evaluate the impact of interventions such as vaccines that aim to reduce disease burden.

To address these surveillance goals, Haiti introduced a number of new surveillance activities that began with building capacity of staff to strengthen VPD surveillance and outbreak response. Since 2012, 3–4 Stop Transmission of Polio field staff (https://www.cdc.gov/globalhealth/immunization/stop/index.htm) were assigned to Haiti every year to support active and case-based surveillance and outbreak response activities. Moreover, an assistant epidemiologist was hired in each department to support departmental epidemiologists in VPD surveillance activities. Multiple trainings for department epidemiologists and immunization staff were conducted to promote a coordinated VPD investigation and outbreak response. The major VPD surveillance activities implemented to improve detection of and response to VPDs in Haiti are mentioned in the following sections.

### Establishment of case-based surveillance and active case search for measles, rubella, and CRS.

A case-based surveillance system is an essential requirement to verify and maintain measles, rubella, and CRS elimination in the Americas.^3^ Therefore, in 2012, MSPP established a case-based surveillance system for AFP, measles, rubella, and CRS, which was integrated within and complementary to the national notifiable disease surveillance system. Department epidemiologists investigated all suspected cases identified and reported, completed individual case investigation forms, and collected specimens that were transferred to the national public health laboratory for testing. Information from the case investigations were entered in a database at the Directorate of Epidemiology, Laboratory and Research, and were also shared with PAHO for its weekly measles, rubella, and polio bulletins.^35^

In addition, active case search for VPDs was established to complement and validate routine reporting under the national notifiable disease surveillance system, which includes over 350 health facilities that passively report diseases on a monthly basis. For the active case search, assistant epidemiologists visited health facilities across each department on a routine and systematic basis determined by patient volume. They reviewed registers and medical records to identify suspected cases of VPDs and determined whether these cases had already been reported and investigated. If the suspected cases had not been investigated previously, assistant epidemiologists followed up and completed a case-based investigation form.

**Figure 2.**
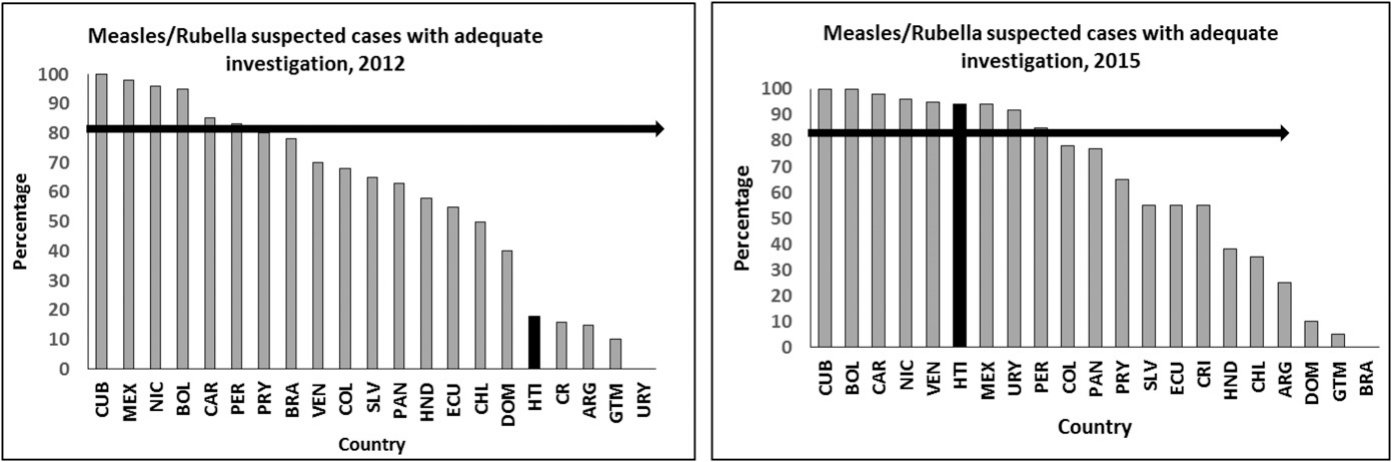
Percentage of measles and rubella suspected cases with adequate investigation in Haiti compared with other countries in Latin America and the Caribbean, 2012 and 2015. Adequate investigation includes at minimum: home visit within 48, hours of notification; completeness of relevant data (i.e., name and/ or identifier, place of residence, sex, age or date of birth, date of reporting, date of investigation, date of rash onset, date of specimen collection, presence of fever, date of prior measles–rubella vaccination and travel history). Source: PAHO.^35^

As a result of those interventions, the quality of VPD surveillance improved significantly within the first year as shown in Table 3. Almost all measles/rubella surveillance performance indicators were well below expected targets during 2010–2012 but they gradually improved to exceed expected targets for all indicators in 2013.^35^ In 2014–2015, the rates of suspected measles and rubella cases were below the required target. However, all other indicators were within acceptable performance. Most notably, compared with other countries in the Latin America and Caribbean region, Haiti moved from being among the fourth worst performing country in 2012, to the sixth best performing country in 2015 for the adequate investigation of suspected cases of measles and rubella (including, at a minimum, home visit within 48 hours of notification and completeness of collection of relevant data: name and/or identifier, place of residence, sex, age or date of birth, date of reporting, date of investigation, date of rash onset, date of specimen collection, presence of fever, date of prior MR vaccination, and travel history) (Figure 2).

**Table 3:** Performance indicators of measles/rubella surveillance in Haiti, 2010–2015

Indicators	Expected results*	2010	2011	2012	2013	2014	2015
Annual rate of measles/rubella (MR) suspected cases	≥ 2 per 100,000 population	0.09	0.2	1.3	3.5	1.1	1.6
Percent of MR cases with adequate investigation†	≥ 80	50	0	17	90	90	92
Percent of MR cases with adequate samples	≥ 80		0	91	98	98	95
Percent of MR cases with sample received at laboratory within 5 days	≥ 80	0	0	46	85	88	78
Percent of MR cases with laboratory results within 4 days	≥ 80	0	0	1	78	75	76

MR = measles–rubella; PAHO = Pan American Health Organization. Source: PAHO.^35^

* Measles and rubella surveillance performance indicators for the PAHO region.^3^

† Adequate investigation includes at minimum, home visit within 48 hours of notification, completeness of relevant data (i.e., name and/or identifier, place of residence, sex, age or date of birth, date of reporting, date of investigation, date of rash onset, date of specimen collection, presence of fever, date of prior MR vaccination, and travel history).

### Establishment of sentinel surveillance for rotavirus and meningitis.

To document the burden of rotavirus diarrhea and meningitis in Haiti and monitor the impact of vaccination on the burden of those diseases among children under 5 years of age, MSPP, with the support of CDC, established four sentinel surveillance sites for rotavirus diarrhea in different areas of the country in 2012.^36^ This surveillance system was established a year before the national introduction of the vaccine to obtain baseline data about rotavirus burden and epidemiology in Haiti. In 2014, surveillance for rotavirus was expanded to include two additional sites. During 2013–2015, the surveillance system has demonstrated consistently increased rotavirus activity in Haiti during the first 3–4 months of the calendar year in children < 5 years old,^36^ a finding that parallels rotavirus activity in other parts of Latin America.^37^ The surveillance system will also provide a platform to evaluate the impact and effectiveness of the vaccine.

The same surveillance system also provided the framework for a new sentinel surveillance system for meningitis, which was launched in 2013 to obtain baseline data on pneumococcal meningitis in Haiti prior to the introduction of PCV-13. In the first year of surveillance from four health facilities, 24% of cerebrospinal fluid samples tested from children presenting with meningitis were positive for *Streptococcus* pneumonia and 16% of the samples were positive for Hib (Laboratoire National de Santé Publique, unpublished data). Currently, efforts are underway to expand and strengthen existing meningitis surveillance to document burden. In addition, to complement meningitis surveillance, a pneumococcal nasopharyngeal carriage study was conducted in 2016 to document circulating serotypes before PCV-13 introduction and to serve as a baseline to measure the impact of the PCV-13 vaccine on the carriage of vaccine-type and non-vaccine type serotypes in the target population and to provide an estimate of vaccine effectiveness.

### Establishment of environmental surveillance for polio.

The use of environmental surveillance (ES) to complement AFP surveillance is a principal activity proposed in the WHO’s Polio Eradication and Endgame Strategic Plan (2013–2018).^20^ In July 2014, in response to the strategic plan, PAHO’s Technical Advisory Group recommended that PAHO propose options for ES in selected settings in the region and urged the implementation of ES toward validating the elimination of WPV and cVDPVs.^38^ Haiti was given particular consideration for ES for VDPVs because of 1) long history of suboptimal polio vaccination coverage, 2) poor sanitary conditions, 3) high level of population movement and high numbers of international visitors, and 4) history of a cVDPV1 outbreak during 2000–2001.^19,21,39^ In 2016, ES was established in two cities—Gonaives and Port-au-Prince—to monitor the emergence of VDPVs and the presence of WPV during 2016–2019, which correspond to the last years that OPV will be used in Haiti. Results generated from the ES will allow immediate detection and response to any polio cases.

## CONCLUSIONS—THE WAY FORWARD

During 2011–2016, Haiti has considerably expanded and strengthened its vaccination services and many of the benefits of these efforts have been documented. The increased capacity of the vaccine cold chain storage and more EVM have enabled more efficient vaccine delivery, and the addition of three new vaccines has the potential to save > 5,000 lives each year. Moreover, the country’s commitment to the elimination of measles, rubella, and CRS through the establishment of case-based surveillance has led to a substantial improvement in measles and rubella surveillance indicators and promoted the verification of elimination of those diseases in Haiti. Haiti has maintained a polio-free status since 2001 and most recently, in 2017, was verified that it has eliminated maternal and neonatal tetanus.

Despite these improvements, more work is needed to improve vaccination coverage and VPD surveillance in Haiti. During 2014–2015, vaccination coverage decreased compared with 2011–2013. Therefore, efforts are needed to prevent vaccine stock-outs and better identify and reach unvaccinated children. A drop in vaccination coverage increases the risk of outbreaks as witnessed during the 2004, 2009, and 2015 diphtheria outbreaks that affected older children and adults who had not been vaccinated prior to 2010.^40,41^ In addition, this situation often invokes the need for campaign-style responses which, although important, further direct limited DPEV resources from their core role in reaching and sustaining high routine vaccination coverage. Moreover, AFP and neonatal tetanus surveillance indicators need to be improved and measles, rubella, and CRS surveillance performance should be maintained.

During 2012–2016, new vaccines were introduced in the routine vaccination schedule (pentavalent, rotavirus, IPV) while other vaccines (oral cholera vaccine) were used in vaccination campaigns. Moving forward, evidence-based information is needed to guide MSPP and DPEV in making decisions regarding the introduction of new vaccines (e.g., birth dose of hepatitis B vaccine, human papilloma virus vaccine, oral cholera vaccine) and endorsing any required changes in the routine vaccination schedule (e.g., best age to introduce a DTP booster dose, second dose of measles vaccine, and implementation of school-based vaccination). Therefore, MSPP and DPEV should consider establishing a National Immunization Technical Advisory Group composed of national multidisciplinary experts responsible for providing independent, evidence-informed advice to health authorities on policy issues related to immunization and vaccines for all age groups.^42,43^

Haiti has been heavily dependent on international donors to purchase vaccines and implement vaccination activities, a dynamic that has only worsened in the postearthquake period due to budget “displacement.” However, postearthquake funding for Haiti is decreasing as international partners address many competing public health emergencies internationally such as Ebola, Zika, and yellow fever outbreaks. Therefore, MSPP should develop a sustainable immunization financing plan to gradually become responsible for purchasing routine vaccines and cofinancing new vaccine procurement. Country ownership and commitment to vaccination is one of the guiding principles of the Global Vaccine Action Plan.^44^ High dependence on external funding, persistently low routine vaccination coverage, and competing vaccination program priorities are potential threats to sustaining the gains achieved thus far. Political commitment at all levels, with favorable economic and legal environments, is needed to maintain and build upon the substantial improvements in the vaccination program during the past 5 years and to continue to strengthen vaccination services and VPD surveillance.
